# Construction and Application of an F1-Derived Doubled-Haploid Population and High-Density Genetic Map for Ornamental Kale Breeding

**DOI:** 10.3390/genes14112104

**Published:** 2023-11-20

**Authors:** Ning Guo, Shuo Han, Mei Zong, Guixiang Wang, Mengmeng Duan, Fan Liu

**Affiliations:** State Key Laboratory of Vegetable Biobreeding, National Engineering Research Center for Vegetables, Beijing Key Laboratory of Vegetable Germplasms Improvement, Key Laboratory of Biology and Genetics Improvement of Horticultural Crops (North China), Beijing Vegetable Research Center, Beijing Academy of Agriculture and Forestry Sciences, Beijing 100097, China; guoning@nercv.org (N.G.); hanshuo@nercv.org (S.H.); zongmei@nercv.org (M.Z.); wangguixiang@nercv.org (G.W.); duanmengmeng@nercv.org (M.D.)

**Keywords:** ornamental kale, F_1_DH population, whole-genome resequencing, high-density genetic linkage map

## Abstract

Ornamental kale (*Brassica oleracea* var. *acephala*) is an attractive ornamental plant with a range of leaf colors and shapes. Breeding new varieties of ornamental kale has proven challenging due to its lengthy breeding cycle and the limited availability of genetic markers. In this study, a F_1_DH ornamental kale population comprising 300 DH lines was constructed using microspore culture. A high-density genetic map was developed by conducting whole-genome sequencing on 150 individuals from the F_1_DH population. The genetic map contained 1696 bin markers with 982,642 single-nucleotide polymorphisms (SNPs) spanning a total distance of 775.81 cM on all nine chromosomes with an average distance between markers of 0.46 cM. The ornamental kale genetic map contained substantially more SNP markers compared with published genetic maps for other *B. oleracea* crops. Furthermore, utilizing this high-density genetic map, we identified seven quantitative trait loci (QTLs) that significantly influence the leaf shape of ornamental kale. These findings are valuable for understanding the genetic basis of key agronomic traits in ornamental kale. The F_1_DH progenies provide an excellent resource for germplasm innovation and breeding new varieties of ornamental kale. Additionally, the high-density genetic map provides crucial insights for gene mapping and unraveling the molecular mechanisms behind important agronomic traits in ornamental kale.

## 1. Introduction

Ornamental kale (*Brassica oleracea* var. *acephala* DC.), as a cultivated variety of *Brassica oleracea* (CC, 2n = 18), is an excellent ornamental foliage plant with a range of leaf colors and shapes. It is widely cultivated as a landscaping plant, potted plant, and for cut foliage. The plants are sufficiently tolerant of frost and chilling that they can grow vigorously in regions that experience low temperatures [1]. The ornamental value and cold tolerance make it a desirable bedding plant in cold seasons and areas. Compared with other *Brassica* crops, fewer varieties of ornamental kale are available commercially and the availability of breeding resources is limited, which has greatly hindered germplasm innovation and the breeding of new varieties.

*B. oleracea* shows obvious heterosis, and cross-breeding is used to produce F_1_ hybrids, which ensures strong uniformity and protection of varietal rights, and is the main method used to breed new ornamental kale varieties. Inbred (true-breeding) lines that are homozygous at virtually all loci enable both consistent production of superior hybrid plants as well as genetic analysis [2,3,4]. Traditional inbreeding, however, requires 6–8 generations of selfing or sib-mating to generate inbred lines, which is labor-intensive and time-consuming. However, the recent technique of isolated microspore culture offers a faster way to produce doubled-haploid (DH) plants, which are essential for consistent hybrid production and genetic analysis [5,6]. Consequently, this technique has been successfully applied to morphotypes of *B. oleracea*, such as cabbage, broccoli, and kale, and large-scale DH lines have been developed and used in breeding [6,7,8].

Populations of DH lines have been used extensively for high-density genetic linkage map construction and quantitative trait locus (QTL) mapping, as they are permanent and completely homozygous. DH lines have clear advantages over F_2_ populations, which consist of individual plants that are often heterozygous. However, the rate of recombination between the parental genomes is lower in DH lines compared to recombinant inbred lines. Creating and maintaining recombinant inbred lines is challenging due to the self-incompatibility of most *B. oleracea* accessions [9]. A DH population provides many homozygous progenies with abundant phenotypic diversity suitable as breeding resources.

Agronomic traits, which are vital for crop improvement, are often complex. Genetic maps, built over the past 30 years for *B. oleracea*, serve as a foundation for studies of QTL mapping and gene fine mapping. Considering all these genetic linkage maps, molecular markers ranging in number from 92 to 4787 were used and the linkage group lengths ranged from 65 to 1738 cM [10]. The first high-density genetic linkage map of *B. oleracea*, with 1257 molecular markers including sequence-related amplified polymorphism and simple sequence repeat (SSR) markers spanning 703 cM in nine linkage groups, was constructed based on a F_2_ population derived from a broccoli and cauliflower cross [11]. The second high-density genetic linkage map was constructed from 1227 markers (602 SSR and 625 single-nucleotide polymorphism (SNP) markers) in nine linkage groups spanning a total of 1197.9 cM with an average of 0.98 cM, using a cabbage DH population. This map also allowed the assembled scaffolds to be anchored to pseudochromosomes [12]. Next-generation sequencing (NGS) technologies have enabled the development of genome-wide methodologies for construction of ultra-high-density genetic linkage maps in different crops, thus allowing placement of candidate loci within several kilobases in a genome [13]. With advances in sequencing technologies, several reference genomes have been generated for different morphotypes of *B. oleracea*, including kale [14], cabbage [15,16,17,18], cauliflower [18,19], and broccoli [20], which greatly facilitates development of a large number of SNP markers for genetic map construction, leading to the improved efficiency of fine mapping. A high-density genetic linkage map of cabbage was constructed with 4103 genotyping-by-sequencing SNP markers in F_2:3_ progenies; this map covered a total genetic distance of 879.9 cM [21]. A genetic map of cauliflower was constructed with 1776 specific locus amplified fragment (SLAF) markers spanning a total genetic length of 890.01 cM with an average marker interval of 0.50 cM [22]. Recently, a genetic map was constructed with 4787 SLAF markers with a mean marker distance of 0.22 cM in a DH population of broccoli, and loci controlling the hollow stem trait were identified in the genetic map [23]. No high-density linkage genetic maps of ornamental kale have been reported, which limits gene mapping and molecular breeding for agronomic traits to some extent.

To generate new breeding materials and map the genes that control important agronomic traits of ornamental kale, an ornamental kale F_1_-derived doubled-haploid (F_1_DH) population derived from parents that differed markedly in leaf shape and color was constructed using microspore culture. Based on this permanent genetic population, a high-density genetic linkage map was constructed with 1696 bin-markers containing 982,642 SNPs via high-throughput whole-genome resequencing. This map covered a total genetic distance of 775.81 cM in nine linkage groups with an average marker interval of 0.46 cM. Furthermore, we identified seven specific quantitative trait loci (QTLs) using a high-density genetic map that have a substantial impact on the leaf shape of ornamental kale. This research will lay a foundation for the proliferation of ornamental kale breeding and the cloning of agronomically important genes.

## 2. Materials and Methods

### 2.1. Parental Materials and F_1_ Progeny Used to Generate the F_1_DH Population

**Figure 1:**
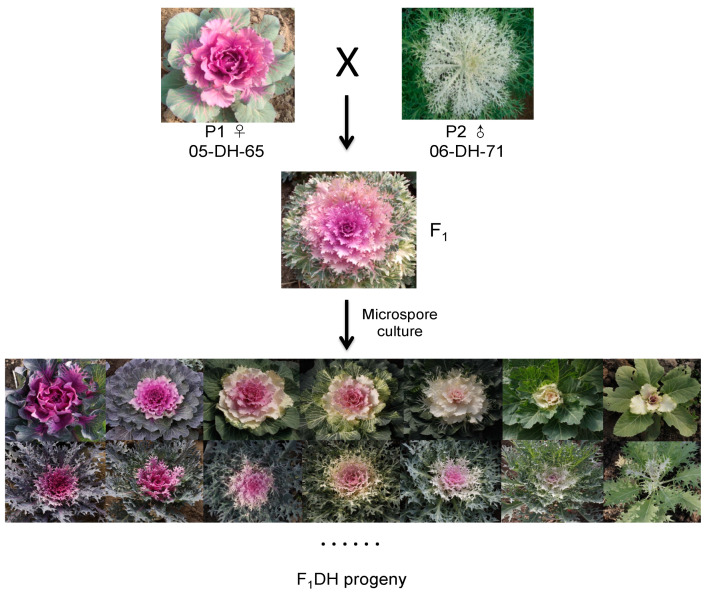
Phenotypes of the parental lines ‘05-DH-65’ (female) and ‘06-DH-71’ (male), the F1 progeny, and different morphotypes among the F_1_DH progeny raised via microspore culture.

Two DH lines, 05-DH-65 (red inner leaves, round leaf, cabbage type) and 06-DH-71 (white inner leaves, lobed leaf, feathered type), which were derived from the commercial cultivars “Paradise Red” and “Coral Sea White” via microspore culture, respectively, were selected as parents to generate the F_1_ hybrid (pink inner leaves, lobed leaf) (Figure 1).

### 2.2. Development of the F_1_DH Population Using Microspore Culture

We conducted microspore culture to obtain DH lines based on a published procedure with minor modifications [8,24,25]. Inflorescences with microspores in the late uninucleate stage were selected for microspore isolation. Buds were excised and subjected to surface sterilization by dipping briefly in 70% ethanol and then soaking in 2% NaOCl for 10 min. Subsequently, the buds were rinsed three times with sterile distilled water for durations of 1, 4, and 10 min, respectively. The buds were then gently macerated in a tube containing NLN13 liquid medium. The microspores were collected via filtration through two layers of 44 µm nylon filters and subsequently washed three times via centrifugation at 100 g for 3–4 min each time. After the final wash, the microspores were resuspended in NLN13 medium at a density of 4 × 10^4^ mL^−1^, and the suspension was poured into Petri dishes for culturing. The cultures were incubated in the dark at 31 °C for 72 h, after which they were transferred to 25 °C, again in the dark. Finally, two to eight embryos from each Petri dish were germinated and rooted on B5-Gamborg medium containing 1% sucrose. Well-rooted plants were then transferred to the glasshouse [25].

### 2.3. Flow Cytometry Analysis

All plants regenerated from microspore culture were analyzed via flow cytometry to identify the ploidy level. About 1.5 cm^2^ young leaf tissue was chopped in nucleus extraction buffer for suspension of intact nuclei [26], and stained with propidium iodide (50 μg/mL) in the dark for 30 min. The samples were analyzed with a flow cytometer (FACSCalibur, BD Biosciences, San Jose, CA, USA) to determine the ploidy. The relative DNA content of the DH parents was adjusted to “channel 100” as the standard. Each histogram was generated from the analysis of at least 10,000 cells for each of four replicates.

### 2.4. Plant Materials for Genetic Map Construction and DNA Extraction

The 150 F_1_DH lines were grown together with the parental lines in the greenhouse of the Beijing Vegetable Research Center (Beijing, China). Young fresh leaves were collected from a single individual and used for DNA extraction using the cetyltrimethyl ammonium bromide method [27]. DNA was quantified with a NanoDrop 2000c spectrophotometer (Thermo Fisher Scientific, Waltham, MA, USA) and was evaluated via electrophoresis in 1.0% agarose gel.

### 2.5. Sequencing Library Construction and High-Throughput Resequencing

After the quality of the DNA samples was checked, the samples were randomly cleaved into 200–500 bp fragments by sonication. Paired-end libraries with insert sizes of ~400 bp were constructed using the NEBNext Ultra DNA Library Prep Kit for Illumina (New England Biolabs, Ipswich, MA, USA) in accordance with the manufacturer’s instructions and sequenced on an Illumina HiSeq 2500 platform (Illumina, San Diego, CA, USA) with the paired-end 2 × 150 bp mode.

### 2.6. SNP Identification and Genotyping

Raw reads from the HiSeq 2500 system were filtered to obtain high-quality reads. The clean reads were mapped to the reference genome. The program used to filter the raw reads was an in-house Perl script from the Biomarker Technology Co. Ltd. (Shunyi, Beijing, China). First, reads with adapter sequences and reads that contained >10% N were filtered, then low-quality reads with >50% of the bases with Q ≤ 10 were removed. To analyze the genomic variation, the clean reads from each sample were counted, and the coverage ratio and distribution of the reads in the TO1000 kale reference genome (https://plants.ensembl.org/Brassica_oleracea/Info/Index?db=core (accessed on 10 May 2019)) [14] were calculated using the Burrows–Wheeler Aligner program [28]. The “CollectInsertSizeMetric” function in the Picard software toolkit (http://broadinstitute.github.io/picard/ (version 2.20.0)) was used to analyze the insert size. The GATK software toolkit (version 4.1.2.0) was used to detect potential SNPs [29]. The Mark Duplicate tool was used to eliminate duplicated reads that may have resulted from PCR amplification. GATK was used for base recalibration, variant calling, and to strictly filter the SNPs to obtain the final SNP clusters [30]. The default parameters were used for SNP calling and mapping for the parental lines and the F_1_DH population. The SNPs identified between the parents were considered polymorphic in the subsequent bin calling. To guarantee the quality of the genetic map, the SNPs were filtered using the following criteria: (1) select markers that are homozygous and inconsistent in the parents; (2) the depth of parental markers is not less than 4×; and (3) remove markers that are not mapped on chromosomes. Gene genotyping of the progenies was based on the parental genotype. Only biallelic SNPs were retained in the final SNP data set. A chi-square test was conducted for bin calling; SNPs that significantly deviated from an extreme segregation distortion (*p* < 0.001) were excluded.

### 2.7. Genetic Map Construction and Evaluation

The HighMap software (http://highmap.biomarker.com.cn/, accessed on 10 May 2019 (version 1.0)) was used to perform linkage grouping, marker ordering, genotyping error correction, and for mapping evaluation [31]. A modified sliding window method was used for bin calling [32]. The SNPs between the adjacent recombinant breakpoints were combined into a recombination bin. The bin markers were used to construct linkage groups with HighMap. The SMOOTH error correction strategy was performed based on the parental contribution of the genotypes [33], and the Kosambi mapping function was used to estimate the map distances. The ALLMAPS program was used to construct the chromosomes by comparing the constructed genetic map and the kale genome map [34]. Haplotype and heat maps were constructed using the “draw haplotype-map.pl” and “draw heatmap.pl” Perl scripts, respectively.

### 2.8. Relationship between the Genetic and Physical Maps

The sequences of all bin markers used to construct the linkage map were aligned to the physical sequences of the TO1000 kale reference genome [14]. Collinearity between genetic and physical positions was determined by plotting genetic marker positions (in centimorgans) against their physical positions (in megabases). The BLAST program was used to confirm their physical positions in the genome. Spearman correlation coefficients were calculated to assess the collinearity between the genetic and physical maps [35].

### 2.9. Phenotyping of Leaf Shape

During the fall of 2021, a group of 150 offspring from the F_1_DH population was cultivated in the open fields at the Beijing Vegetable Research Center. This cultivation was replicated three times biologically to document traits associated with leaf shape. The parameters recorded included leaf length (measured from the base of the petiole to the leaf blade tip), leaf width (measured at the leaf’s broadest point), and the leaf shape index (the ratio of length to width). To analyze these data, we utilized GraphPad Prism 9 software (version 9.3) to conduct frequency analysis on the gathered and computed values for leaf length, width, and shape index, which was then visualized through histogram representations.

### 2.10. QTL Analysis

The analysis of QTL (Quantitative Trait Loci) involved the use of R/qtl software (version 1.41-6). To identify significant cofactors, an automatic selection process (reverse elimination, with a significance threshold of *p* < 0.05) was applied. The LOD (Logarithm of Odds) significance threshold, based on 1000 permutations, was used to determine the statistical significance level. The location of each QTL was determined by identifying the peak LOD score and examining the surrounding region. The percentage of phenotypic variation explained by each QTL (R2) was estimated at the highest probability peak. For the annotation of candidate genes, the TO1000 kale genome available at https://plants.ensembl.org/Brassica_oleracea/Info/Index?db=core (accessed on 10 May 2019) was consulted.

## 3. Results

### 3.1. Microspore Embryogenesis of Ornamental Kale F1 Progeny

**Figure 2:**
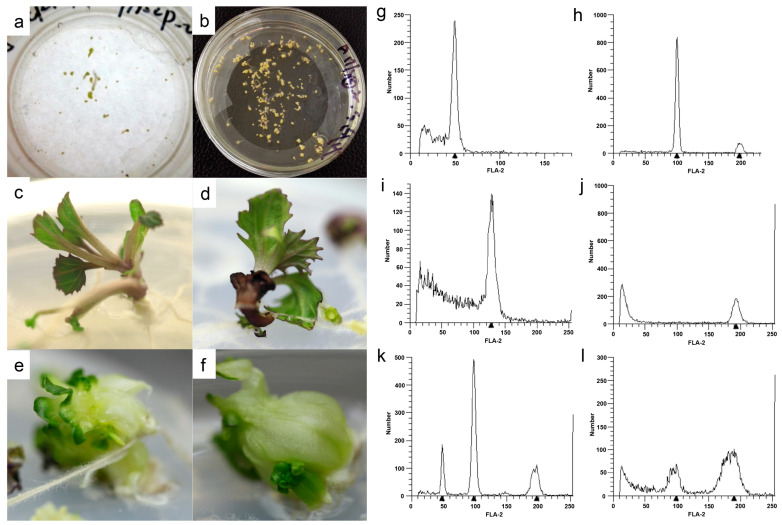
Embryoid formation and plant regeneration via microspore culture, and ploidy detection among regenerated plants derived from the ornamental kale F_1_ progeny. Globular, heart, torpedo, and cotyledon-stage embryos in the same Petri dish (**a**). Episomal embryoids from microspore culture (**b**). Regenerated shoots directly generated from embryoids (**c**,**d**). Adventitious buds formed secondary embryos through somatic embryogenesis (**e**,**f**). Flow cytometry was used for ploidy detection among the regenerated plants (**g**–**l**).

In this research, the F_1_ progeny generated by crossing two DH lines, with strong differences in leaf shape and color (Figure 1), showed a high capability of producing embryos and developing into plants. The first cell division occurred on the fourth day after microspore culture. Cultured cells continued to divide to form, in turn, a cell group, original embryo, globular embryo, heart-shaped embryo, torpedo, and the cotyledon-stage embryo, which appeared after approximately 25 days. The differentiation and development of microspore cells were weakly synchronized in each Petri dish; even one month later, the embryoids in the same Petri dish were at different developmental stages (Figure 2a). Regenerated plants were formed from the episomal embryoids (Figure 2b) via different pathways. Some embryoids developed directly into regenerated plants (Figure 2c,d), whereas others formed secondary embryos through somatic embryogenesis of the hypocotyl, and then underwent further differentiation via secondary embryogenesis to generate adventitious buds that formed regenerated plants (Figure 2e,f).

### 3.2. Ploidy Level and Spontaneous Chromosome Doubling

In total, 1026 regenerated plants were obtained from microspore culture of the ornamental kale F_1_ progeny. Their ploidy levels were determined via flow cytometry. The regenerated plants comprised 238 haploids (n, Figure 2g), 608 diploids (2n, Figure 2h), 6 triploids (3n, Figure 2i), 97 tetraploids (4n, Figure 2j), and 77 mixed-ploidy plantlets consisting of 55 “n + 2n” plantlets (Figure 2k) and 22 “2n + 4n” plantlets (Figure 2l). The relative proportions of diploids, haploids, triploids, tetraploids, “n + 2n”, and “2n + 4n” plantlets were 23.21%, 59.26%, 0.58%, 9.45%, 5.36%, and 2.14%, respectively. The total percentage of spontaneous chromosome doubling (comprising diploids, polyploids, and mixed-ploidy plantlets) was 76.79%.

### 3.3. Construction of the F_1_DH Mapping Population

From the 608 diploids, 300 genotypes that showed phenotypic variation were selected for construction of a F_1_DH population of ornamental kale. The leaf color and leaf shape were investigated when plants were well colored. The colors of colored (inner) leaves of different F_1_DH genotypes formed a continuum from white (no anthocyanin) to purple (more anthocyanin content), and the leaf shapes varied from entire (round) to deeply lobed (feather) (Figure 1). We self-pollinated these DH lines to obtain stable genetic progeny populations, and finally selected 150 inbred lines to form a mapping population for subsequent high-throughput whole-genome resequencing (WGR), genotyping, and high-density genetic map construction.

### 3.4. Whole-Genome Resequencing of the F_1_DH Mapping Population and Two Parents

We performed WGR of the 150 F_1_DH individuals and their two parents on an Illumina HiSeq sequencing platform. The raw reads were filtered, and low-quality sequences and redundant and unpaired reads were removed. In total, 145.12, 72.90, and 694.71 million clean reads were obtained for the parents ‘05-DH-65’ and ‘06-DH-71’ and the 150 F_1_DH progenies, respectively, including 43.45, 21.83, and 208.09 Gb clean data with Q30 percentages of 91.45%, 85.59%, and 90.88%, respectively (Table 1). The respective GC contents were 37.59%, 36.74%, and 37.07%, and were normally distributed (Table 1 and Supplementary Figure S1). These results indicated that the sequencing data for the parents and the 150 F_1_DH individuals were of good quality.

The clean reads were aligned to the kale-like rapid cycling reference genome (TO1000) [14] using the Burrows–Wheeler Aligner (BWA) software (version v0.7.8) [28]. In total, 94.12% and 92.59% of the total clean reads were mapped for the parents ‘05-DH-65’ and ‘06-DH-71’, respectively, and the average mapping rate for the F_1_DH individuals was 93.55%. The percentages of mapped reads of the two parents and the average mapped reads of the progeny were all higher than 90%, and the properly mapped ratio (on average) was 80.85% and 78.45% for the two parents and 81.41% for the progeny (Table 2). The average coverage depth was 64× and 34× for the two parents, and 2.6× for the progeny. There were more than 90% reads with coverage higher than 5× for the two parents, and more than 70% reads with average coverage higher than 1× for the progeny (Supplementary Table S1). The even distribution of read coverage depth on the nine chromosomes indicated that the genome resequencing was sufficiently random (Supplementary Figure S2).

### 3.5. SNP Calling and High-Density Bin Genetic Linkage Map Construction

Based on the genotype data for the two parents, SNP markers were identified. In total, 1,779,215 SNPs were detected between the two parental lines. Among these SNPs, 1,491,165 (83.8%) SNPs were distributed on the nine pseudochromosomes and 288,050 SNPs were on unanchored scaffolds. The SNPs were distributed throughout the nine pseudochromosomes, with the highest number of SNPs occurring on chromosome C03 and the fewest on chromosome C06 (Supplementary Figure S3). To ensure the quality of the map, the markers were filtered and screened (see Section 2). After filtering, 1,007,400 SNPs were able to genotype the F_1_DH population and were polymorphic. All of the SNP sites in the F_1_DH population were integrated into a recombination bin unit, and 1781 recombinant bins were used to construct the genetic map. To the best of our knowledge, the SNP markers that were mapped are more numerous than for previously reported genetic maps of *B. oleracea*, and form the first high-density genetic map of ornamental kale generated by WGR.

**Figure 3:**
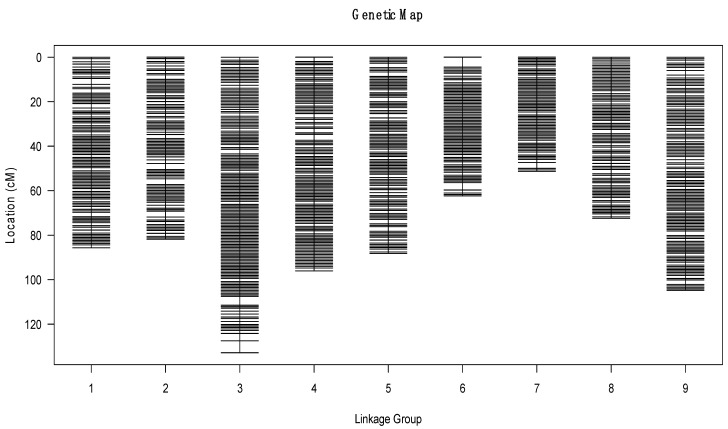
High-density genetic map of ornamental kale constructed from bin markers based on whole-genome resequencing. The x-axis presents the linkage group and the y-axis presents the genetic distance.

To ensure the quality of the genetic map, we further polished and filtered the bins. The final high-density genetic map consisted of nine chromosomes and was based on 1696 recombination bins containing 982,642 SNPs with a well-distributed distance (Figure 3). The length of the nine linkage groups ranged from 51.34 cM to 132.88 cM, and the total distance of the genetic linkage map was 775.81 cM, with an average distance of 0.46 cM between adjacent bin markers. The number of bin markers on the different chromosomes ranged from 126 to 272, and the average length of the bins was 0.295 Mb (Table 3).

In general, the bin markers were well distributed on the genome and more than 99% of the intervals between adjacent markers were less than 5 cM. The largest gap, which occurred in LG3, was only 5.35 cM. LG3 was the largest linkage group, which covered 132.88 cM and included 272 bin markers, with an average distance of 0.49 cM between adjacent markers. The smallest LG was LG7, which covered 51.34 cM and included 126 bin markers with an average distance of 0.41 cM between adjacent markers (Table 3).

Haplotype and heat maps were used to assess the quality of the genetic map. The haplotype map, which counted the haplotype source in all linkage groups, reflected the double crossovers of the population and deletions, indicating recombination events and marker ordering errors. Haplotype maps were generated for each of the 150 lines in the F_1_DH population as well as the parental lines. As illustrated in Supplementary Figure S4, almost all of the recombination blocks were clearly defined.

The genetic map is essentially a multi-locus recombination analysis. The recombination rate is lower if the marker is closer. The linkage relationship of the markers in the linkage group can be reflected by drawing heatmaps of marker relationships. Heatmaps of the nine linkage groups were generated separately based on pair-wise recombination values for the 1696 recombination bin markers. The linkage relation heatmap provides a visual representation of the relationship between recombination markers on a single chromosome, allowing for the identification of potential marker ordering errors (Supplementary Figure S5). The heatmap utilizes colors to indicate the recombination rates between markers, with yellow indicating lower recombination rates and purple indicating higher rates. The heatmap analysis demonstrated that the construction of the genetic map was accurate, as the linkage groups were clearly defined and easy to visualize.

To evaluate the collinearity between the genetic map and the kale reference genome (TO1000), all bin markers were mapped to the reference genome. As shown in Supplementary Figure S6, the relationship between the genetic and physical maps were generally linear for all nine chromosomes, which indicated that the genetic map was consistent with the physical map. The Spearman correlation coefficients between the genetic and physical positions of the nine linkage groups were more than 0.96 (Supplementary Table S2). These results showed that these linkage groups show high levels of genetic collinearity with the physical map. The high collinearity suggested that the markers accurately cover the nine chromosomes and that they sufficiently cover the reference genome.

### 3.6. QTL Analysis for Leaf Shape Traits of Ornamental Kale

**Figure 4:**
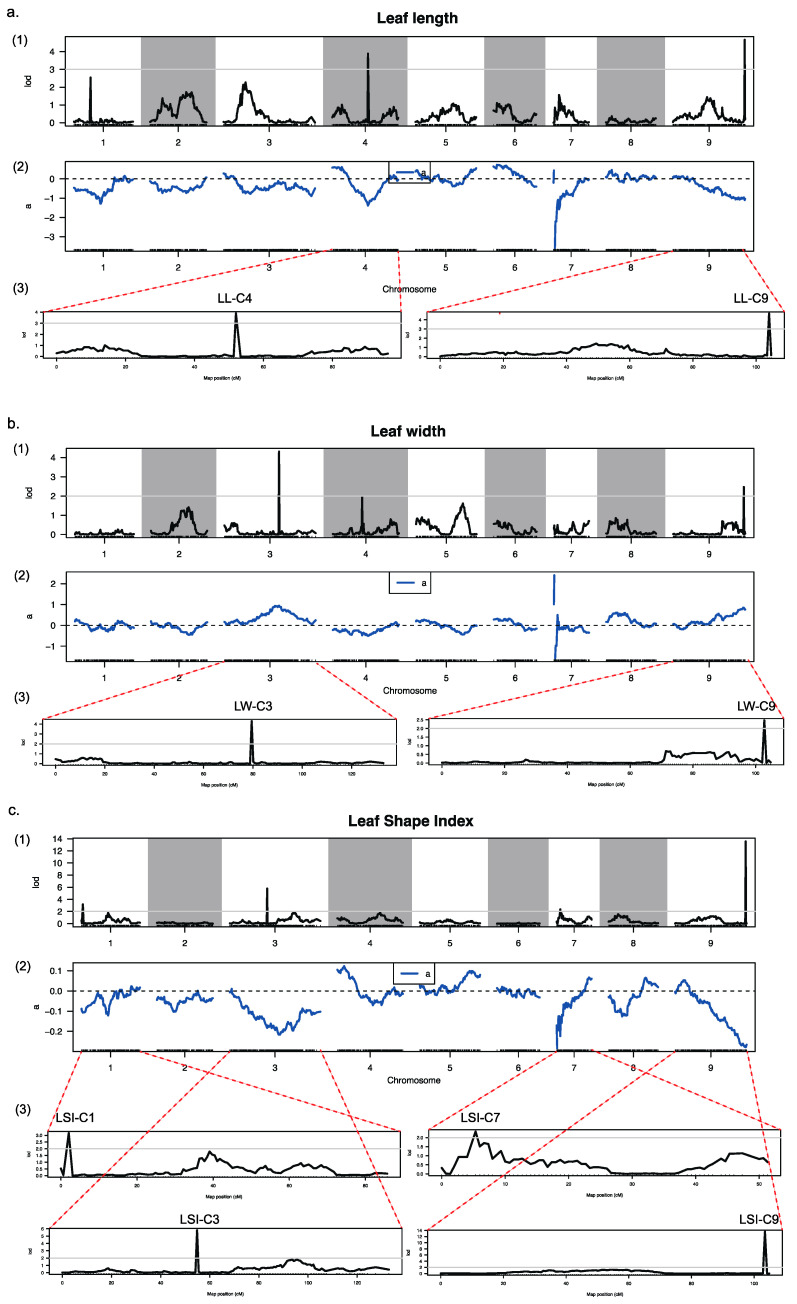
Locations of leaf-shape-related traits on genetic linkage map. (**a1**) LOD score for the variation in the leaf length along nine LGs. (**a2**) Phenotypic contribution rate along the nine LGs for variation of the leaf length. (**a3**) LOD scores for the variation in the leaf length along the fourth (C4) and ninth (C9) LGs. (**b1**) LOD score for the variation in the leaf width along nine LGs. (**b2**) Phenotypic contribution rate along the nine LGs for variation of the leaf width. (**b3**) LOD scores for the variation in the leaf width along the third (C3) and ninth (C9) LGs. (**c1**) LOD score for the variation in the leaf shape index along nine LGs. (**c2**) Phenotypic contribution rate along the nine LGs for variation of the leaf shape index. (**c3**) LOD scores for the variation in the leaf shape index along the first (C1), third (C3), seventh (C7) and ninth (C9) LGs. In (**a1**,**a3**,**b1**,**b3**,**c1**,**c3**), the horizontal ordinate presents the order of the markers in the linkage group; the vertical ordinate presents the LOD values; curves in the plot indicate the genetic coordinate and the LOD score of the detected QTL; the gray line indicates the threshold; the area above the threshold is the associated QTL area. In (**a2**,**b2**,**c2**), the horizontal ordinate presents the order of the markers in the linkage group; the vertical ordinate presents the contribution rate; ‘a’ (the blue curve) indicates the additive effectvalue corresponding to the marker.

One of the main applications of high-density genetic maps is to conduct QTL map-ping studies for important agronomic traits. The leaf shape variation of ornamental kale is significantly different, ranging from flat round to elongated oval. The leaf shape is one of the important agronomic traits that breeders focus on when breeding ornamental kale. In our measurements, we assessed the leaf length (LL), leaf width (LW), and computed the leaf shape index (LSI, defined as the ratio of LL to LW). Our analysis indicates that for the F_1_DH group, the distribution of LL, LW, and LSI adheres to a normal or near-normal distribution (Supplementary Figure S7). Using the high-density genetic map along with phenotypic data, we examined QTLs associated with leaf shape characteristics. This examination led to the discovery of two QTLs influencing LL on chromosomes C4 and C9, as depicted in Figure 4a (denoted as LL-C4 and LL-C9), two QTLs affecting LW on chromosomes C3 and C9, as illustrated in Figure 4b (LW-C3 and LW-C9), and four QTLs for LSI on chromosomes C1, C3, C7, and C9, as shown in Figure 4c (LSI-C1, LSI-C3, LSI-C7, and LSI-C9) (Figure 4; Table 4).

## 4. Discussion

### 4.1. Development of DH Lines Is an Effective Method for Germplasm Innovation of Ornamental Kale

The development of DH lines is an effective approach for germplasm innovation in plant breeding. The DH lines are genetically uniform and are true-breeding lines, and thus have the same genetic constitution as the original haploid plant from which they were derived. As a result, they are useful for the development of new cultivars with desirable traits, such as disease resistance, improved quality, and higher yield. The DH lines can be generated by several methods, including microspore culture, anther culture, and ovule culture. These methods allow for the rapid production of genetically uniform DH lines, which can be used to improve the efficiency of plant breeding programs [5]. By using DH lines, plant breeders can reduce the time required by traditional breeding methods, which can take several years. The DH lines can be used to evaluate and select desirable traits in a shorter period, allowing breeders to quickly develop new cultivars with improved characteristics. 

Ornamental kale is a popular plant on account of its colorful foliage and unique appearance, but compared with other brassicaceous crops, limited germplasm resources are available for this plant. This can limit the potential for development of new and improved cultivars with desirable traits. Breeding ornamental kale can be challenging owing to its complex genetics and reproductive characteristics. This can make it difficult to develop new cultivars or to transfer desirable traits between varieties.

In this study, we developed a stable and efficient microspore culture system for ornamental kale to produce double haploids. The regenerated plants can be generated by different pathways from embryoids. The spontaneous chromosome-doubling rate was relatively high (>75%) during microspore regeneration, and the percentage of diploids was more than 20%. The F_1_DH progenies exhibited rich phenotypic diversity, especially in leaf shape and color, and represented novel phenotypes that were not present in the parents. These results indicated that development of DH lines by microspore culture is an effective method for germplasm innovation of ornamental kale.

### 4.2. The F_1_DH Population Will Play Important Roles in Genetic Analysis of Ornamental Kale

The F_1_DH population is a type of mapping population that is commonly used in genetic map construction and QTL analysis. The F_1_DH lines are homozygous at all loci and hence are ideal for genetic mapping and QTL analysis. This enables accurate and consistent mapping of genetic loci and precise identification of QTLs [4]. The F_1_DH lines can be derived from F_1_ hybrids that possess desirable traits, allowing for the efficient transfer of these traits to the mapping population. This is particularly useful in plant breeding, where desirable traits can be selected and incorporated into new varieties [36]. The homozygosity of F_1_DH lines allows for the generation of high-density genetic maps and precise identification of QTLs. This is particularly useful in QTL analysis, where high-resolution mapping is required to identify the genetic loci responsible for complex traits [37].

Ornamental kale exhibits conspicuous variation in leaf color and shape, and is a suitable material to reveal the genes responsible. In this study, two ornamental kale DH lines differing markedly in leaf color and shape (one parent with red round leaves and the other parent with white feather leaves) were used as the parents and 300 diploid individuals were selected to construct the F_1_DH population. The F_1_DH progenies represented abundant genetic diversity in leaf color and shape, which exhibited a continuum from white to purple and from deeply lobed to entire. The F_1_DH population provides an excellent genetic resource and will shorten the time required for breeding new varieties, and also lays the foundation for mapping the genes that control leaf color and shape in ornamental kale. 

### 4.3. Features of the High-Density Genetic Map of Ornamental Kale

A genetic map, which is a representation of the location of genes, genetic markers, and other genetic features on a chromosomes or genome, is important for finding loci of interest, fine mapping, gene identification, map-based cloning, comparative genomics, genome assembly, and MAS breeding [13,38]. The rapid development of NGS technologies, and the publication of the kale genome [14] and several other high-quality *B. oleracea* reference genomes [16,17,18,19,20], have made it possible to construct a high-density genetic map of ornamental kale using SNP markers and accurate genotyping. Several NGS-based genetic maps for *B. oleracea* have been published in recent years [21,22,23,39]. However, these genetic maps all used simplified genome sequencing, such as genotyping via sequencing or SLAF markers. There are few reports of *B. oleracea* genetic maps based on WGR and no high-density genetic maps of ornamental kale have been reported previously. This sequence-based genotyping method is faster and more accurate than marker-based genotyping methods for constructing a genetic map and detecting recombination breakpoints [32,40]. The WGR mapping method significantly improves the efficiency of QTL mapping and marker development [41]. Based on the WGR method, 982,642 SNP markers have been mapped on the genetic map. Many more SNP markers were mapped to the genetic map than in previous studies on other *B. oleracea* crops [21,22,23], which will provide valuable tools for future candidate-gene identification, map-based gene cloning, and MAS.

In this study, we sequenced both parental lines at a high coverage depth and sequenced the F_1_DH offspring at a low coverage depth. A prominent feature of the WGR method for construction of genetic maps that was used in the present research is that it integrates SNP discovery, SNP validation, and genotyping [35,42,43]. We sequenced parental lines with more than 30-fold sequencing depth and each F_1_DH progeny with an average 2.6-fold sequencing depth, which was sufficient to detect the recombination breakpoints. We obtained 43.45, 21.83, and 208.09 Gb of high-quality clean reads from the female parent, the male parent, and their progenies, respectively. By implementing rigorous analysis criteria, recombination intervals were removed, and bin markers were created using precise genotypic data to develop a high-density genetic map. The outcomes revealed that the WGR approach is an effective method for discovering markers and constructing linkage maps with high density. By using the WGR mapping technique, a vast number of genome-wide SNPs were identified that accurately represent the genomic and genetic diversity characteristics, and provided an abundance of polymorphisms for constructing the map [44,45].

In total, 1696 recombination bin markers representing 982,642 SNP markers were mapped to nine linkage groups. The total length of the linkage map was 775.81 cM, with an average distance of 0.46 cM between adjacent bin markers. The collinearity of the genetic and physical maps was generally uniform for all nine chromosomes, which suggested that the markers accurately cover the chromosomes. Visual evaluation of the haplotype and heat maps of the genetic map suggested that the F_1_DH population was suitable for genetic analysis. These results indicated that the high-density genetic map of ornamental kale contained a high marker density and was accurately constructed.

### 4.4. Utilizing High-Density Genetic Linkage Map for Leaf Morphology QTL Research

In the field of plant genetics, the use of high-density genetic linkage maps is a key method for examining the quantitative trait loci (QTLs) related to leaf morphology. Leaf shape stands as a crucial horticultural characteristic for ornamental kale, significantly influencing its market value. It is, therefore, a significant subject within both academic research and practical breeding programs. The high-density genetic map is essential for identifying the exact regions within the genome that account for the wide variety in leaf shapes and sizes, attributes that are essential to the ornamental appeal of the plant. Our research focused on evaluating the dimensions and configuration of ornamental kale leaves by measuring leaf length, width, and calculating the leaf shape index. Through detailed QTL mapping, we discerned multiple loci associated with these traits: two for leaf length on chromosomes C4 and C9; three for leaf width on chromosomes C3 and C9; and four for the leaf shape index on chromosomes C1, C3, C7, and C9. Of particular interest is the cluster of three QTLs affecting leaf shape located closely together on chromosome C9. The findings from this QTL localization not only exemplify the practical utility of high-density genetic linkage maps but also establish a groundwork for future research into the critical regulatory genes that determine leaf shape in ornamental kale. This could lead to the creation of molecular markers tightly linked to these traits and support molecular-assisted breeding strategies. Understanding the genetic underpinnings of leaf morphology is vital for enhancing the ornamental kale’s aesthetic quality as well as its agricultural traits like light capture and water loss management. The precise genetic mapping that these tools offer assists in the development of targeted breeding schemes, which aim to produce plant varieties with optimal leaf traits for both decorative and agricultural productivity purposes.

### 4.5. The Application Prospects of the High-Density Genetic Map of Ornamental Kale

The high-density genetic map of ornamental kale can be applied in multiple areas of research and has excellent application prospects. It can be used in genome assembly and annotation to improve accuracy by providing a scaffold for the assembly and identification of gaps or errors. It can be also used in comparative genomics to compare the genetic organization and evolution of different varieties of *B. oleracea* or other species of *Brassica*, which may provide insights into the mechanisms of speciation and genome rearrangement. Moreover, it can be used in QTL mapping to identify QTLs that control complex traits, such as leaf shape, leaf color, and disease resistance, in ornamental kale. It will also play important roles in the development of molecular breeding strategies for ornamental kale improvement, including MAS breeding, genomic selection, and gene editing. Overall, the high-density genetic map of ornamental kale is a powerful tool for genetic analysis and breeding, and its application is likely to continue to expand with continuing advances in genomic technologies.

## 5. Conclusions

In this study, a F_1_DH population of ornamental kale was successfully constructed using microspore culture. The population was used to create a high-density genetic map, which contained a total of 1696 recombination bin markers with an average marker density of 0.46 cM. Using this high-density genetic linkage map, we effectively pinpointed the QTLs associated with the leaf morphology of ornamental kale. These F_1_DH progenies represent an excellent resource for germplasm innovation and the breeding of new varieties of ornamental kale. The high-density genetic map provides useful information for gene mapping and revealing the molecular genetic mechanism of important agronomic traits of ornamental kale. The present results provide valuable insights into the genetic basis of important traits in ornamental kale and provide a valuable resource for breeding and future research.

## Figures and Tables

**Table 1:** Statistics for the whole-genome resequencing data.

Sample	Total Clean Reads	Total Clean Bases	Q30 Percentage (%)	GC Percentage (%)
05-DH-65	145,119,251	43,453,720,350	91.45	37.59
06-DH-71	72,896,717	21,830,253,446	85.59	36.74
F_1_DH offspring	694,710,016	208,094,995,584	90.88	37.07
Total	912,725,984	273,378,969,380	90.84	37.07

**Table 2:** Statistics for the mapping results.

Sample	Clean Reads	Mapped (%)	Properly Mapped (%)
05-DH-65	145,119,251	94.12	80.85
06-DH-71	72,896,717	92.59	78.45
F_1_DH offspring (average)	9,199,180	93.55	81.41

**Table 3:** Distribution of genetic markers on the high-density genetic map.

LG ID	Total Bin Marker	Total Distance (cM)	Average Distance (cM)	Max Gap (cM)	Gaps < 5 cM (%)
LG1	156	85.73	0.55	2.67	100
LG2	164	81.89	0.5	2.73	98.36
LG3	272	132.88	0.49	5.35	99.73
LG4	221	96.06	0.43	2.67	98.89
LG5	179	88.25	0.49	2	100
LG6	170	62.4	0.37	3.34	100
LG7	126	51.34	0.41	2.67	100
LG8	180	72.45	0.4	2	100
LG9	228	104.81	0.46	0.02	100
Total	1696	775.81	0.46		99.66

**Table 4:** Loci associated with leaf length, leaf width, and leaf shape index.

Name	Chromosome	Marker Interval	Position (cM)	LOD	Additive	expl%
LL-C4	C4	Block1663–Block1708	52.01	3.89	−1.39	14.12
LL-C9	C9	Block4146	104.141	4.67	−1.10	8.88
LW-C3	C3	Block1065	79.506	4.31	0.95	15.39
LW-C9	C9	Block4118–Block4144	102.807	2.47	0.85	12.22
LSI-C1	C1	Block314	2.07	3.16	−0.11	4.89
LSI-C3	C3	Block1168	54.838	5.79	−0.18	14.20
LSI-C7	C7	Block2891–Block2970	5.334	2.34	−0.22	21.08
LSI-C9	C9	Block4145	103.474	13.55	−0.28	32.82

Additive: Additive effect value of QTL analysis. expl%: Contribution rate of the QTL locus.

## Data Availability

The SNP genotyping dataset of the parents and progenies of the ornamental kale F_1_DH population is available in the European Variation Archive (EVA) at EMBL-EBI under accession number PRJEB61601 (https://www.ebi.ac.uk/eva/?eva-study=PRJEB61601 (accessed on 10 May 2023)).

## Supplementary Materials

The following are available online at https://www.mdpi.com/article/10.3390/genes14112104/s1, Figure S1: Base ratio distribution for the female parental line 05-DH-65; Figure S2: Coverage depth distribution on chromosomes of the female parental line. The *x*-axis represents the position on each chromosome. The *y*-axis presents the log_2_ value of the coverage depth; Table S1: Coverage depth and coverage ratio of the sequencing data; Table S2: Spearman correlation coefficients between the genetic and physical positions of each linkage group (LG); Figure S3: Heatmap of the distribution of SNPs across the nine chromosomes of the ornamental kale genome. A window size of 0.1 Mb was selected on the genome to count the number of SNPs. The color ranges from green to red, indicating the density of SNPs from low to high; Figure S4: Haplotype map of the genetic map. Each row represents a marker, arranged in the order in the linkage map. Each column represents one sample. Red represents the female parent, blue represents the male parent, and gray indicates missing data. Where the color changes in a column is the location of a recombination event; Figure S5: Heatmap of the genetic map. The x and y axes represent markers. The order of markers in a row and column are arranged according to their genetic distance. Each row and column is arranged in the order of markers on the map. Each cell represents the recombination rate between two markers, with the color gradient from yellow to red to purple indicating a progression from lower to higher recombination rates. Markers that are closer to each other have a lower recombination rate and are closer to yellow in color, while markers that are further apart have a higher recombination rate, indicated by a color closer to purple; Figure S6: Relationship between genetic and physical positions for each chromosome. The x-axis represents the genetic distance on the genetic map, and the y-axis represents the physical position on the genome. The different colors only indicated the different LGs; Figure S7: Histograms depicting the distribution of leaf morphological traits in a plant population. a, b, c, display the leaf length, leaf width, leaf shape index distribution, respectively.

## References

[1] Li Y., Yu X. (2006). Pollination with laser-irradiated pollens breaks cross-incompatibility between zicaitai (*Brassica campestris* var. *purpurea*) and ornamental kale (*Brassica oleracea* var. *acephala*) to produce hybrids with the aid of ovule culture. Sci. Hortic..

[2] Forster B.P., Heberle-Bors E., Kasha K.J., Touraev A. (2007). The resurgence of haploids in higher plants. Trends Plant Sci..

[3] Dunwell J.M. (2010). Haploids in flowering plants: Origins and exploitation. Plant Biotechnol. J..

[4] Forster B.P., Thomas W.T. (2010). Doubled haploids in genetics and plant breeding. Plant Breed. Rev..

[5] Ferrie A., Caswell K. (2011). Isolated microspore culture techniques and recent progress for haploid and doubled haploid plant production. Plant Cell Tissue Organ Cult. (PCTOC).

[6] Zhang W., Fu Q., Dai X., Bao M. (2008). The culture of isolated microspores of ornamental kale (*Brassica oleracea* var. *acephala*) and the importance of genotype to embryo regeneration. Sci. Hortic..

[7] Yuan S., Liu Y., Fang Z., Yang L., Zhuang M., Zhang Y., Sun P. (2011). Effect of combined cold pretreatment and heat shock on microspore cultures in broccoli. Plant Breed..

[8] Lv H., Wang Q., Yang L., Fang Z., Liu Y., Zhuang M., Zhang Y., Yang Y., Xie B., Wang X. (2014). Breeding of cabbage (*Brassica oleracea* L. var. *capitata*) with fusarium wilt resistance based on microspore culture and marker-assisted selection. Euphytica.

[9] Lou P., Zhao J., He H., Hanhart C., Pino Del Carpio D., Verkerk R., Custers J., Koornneef M., Bonnema G. (2008). Quantitative trait loci for glucosinolate accumulation in *Brassica rapa* leaves. New Phytol..

[10] Hirani A.H., Li G. (2021). Genetic Mapping, Quantitative Trait Analysis, and Gene Cloning in *Brassica oleracea*. The Brassica oleracea Genome.

[11] Gao M., Li G., Yang B., Qiu D., Farnham M., Quiros C. (2007). High-density *Brassica oleracea* linkage map: Identification of useful new linkages. Theor. Appl. Genet..

[12] Wang W., Huang S., Liu Y., Fang Z., Yang L., Hua W., Yuan S., Liu S., Sun J., Zhuang M. (2012). Construction and analysis of a high-density genetic linkage map in cabbage (*Brassica oleracea* L. var. *capitata*). BMC Genom..

[13] Jaganathan D., Bohra A., Thudi M., Varshney R.K. (2020). Fine mapping and gene cloning in the post-NGS era: Advances and prospects. Theor. Appl. Genet..

[14] Parkin I.A., Koh C., Tang H., Robinson S.J., Kagale S., Clarke W.E., Town C.D., Nixon J., Krishnakumar V., Bidwell S.L. (2014). Transcriptome and methylome profiling reveals relics of genome dominance in the mesopolyploid *Brassica oleracea*. Genome Biol..

[15] Liu S., Liu Y., Yang X., Tong C., Edwards D., Parkin I.A., Zhao M., Ma J., Yu J., Huang S. (2014). The *Brassica oleracea* genome reveals the asymmetrical evolution of polyploid genomes. Nat. Commun..

[16] Cai X., Wu J., Liang J., Lin R., Zhang K., Cheng F., Wang X. (2020). Improved *Brassica oleracea* JZS assembly reveals significant changing of LTR-RT dynamics in different morphotypes. Theor. Appl. Genet..

[17] Lv H., Wang Y., Han F., Ji J., Fang Z., Zhuang M., Li Z., Zhang Y., Yang L. (2020). A high-quality reference genome for cabbage obtained with SMRT reveals novel genomic features and evolutionary characteristics. Sci. Rep..

[18] Guo N., Wang S., Gao L., Liu Y., Wang X., Lai E., Duan M., Wang G., Li J., Yang M. (2021). Genome sequencing sheds light on the contribution of structural variants to *Brassica oleracea* diversification. BMC Biol..

[19] Sun D., Wang C., Zhang X., Zhang W., Jiang H., Yao X., Liu L., Wen Z., Niu G., Shan X. (2019). Draft genome sequence of cauliflower (*Brassica oleracea* L. var. *botrytis*) provides new insights into the C genome in *Brassica* species. Hortic. Res..

[20] Belser C., Istace B., Denis E., Dubarry M., Baurens F.-C., Falentin C., Genete M., Berrabah W., Chèvre A.-M., Delourme R. (2018). Chromosome-scale assemblies of plant genomes using nanopore long reads and optical maps. Nat. Plants.

[21] Lee J., Izzah N.K., Choi B.-S., Joh H.J., Lee S.-C., Perumal S., Seo J., Ahn K., Jo E.J., Choi G.J. (2016). Genotyping-by-sequencing map permits identification of clubroot resistance QTLs and revision of the reference genome assembly in cabbage (*Brassica oleracea* L.). DNA Res..

[22] Zhao Z., Gu H., Sheng X., Yu H., Wang J., Huang L., Wang D. (2016). Genome-wide single-nucleotide polymorphisms discovery and high-density genetic map construction in cauliflower using specific-locus amplified fragment sequencing. Front. Plant Sci..

[23] Yu H., Wang J., Zhao Z., Sheng X., Shen Y., Branca F., Gu H. (2019). Construction of a high-density genetic map and identification of loci related to hollow stem trait in broccoli (*Brassic oleracea* L. *italica*). Front. Plant Sci..

[24] Custers J., Cordewener J., Fiers M., Maassen B., van Lookeren Campagne M., Liu C. (2001). Androgenesis in *Brassica*: A model system to study the initiation of plant embryogenesis. Curr. Trends Embryol. Angiosperms.

[25] Han S., Guo N., Zhang Y., Zong M., Wang G., Liu F. (2018). Researches on the double haploid breeding of ornamental kale (*Brassica oleracea* var. *acephala*). J. Agric. Biotechnol..

[26] Dpooležel J., Binarová P., Lcretti S. (1989). Analysis of nuclear DNA content in plant cells by flow cytometry. Biol. Plant..

[27] Murray M., Thompson W. (1980). Rapid isolation of high molecular weight plant DNA. Nucleic Acids Res..

[28] Li H., Durbin R. (2009). Fast and accurate short read alignment with Burrows–Wheeler transform. Bioinformatics.

[29] DePristo M.A., Banks E., Poplin R., Garimella K.V., Maguire J.R., Hartl C., Philippakis A.A., Del Angel G., Rivas M.A., Hanna M. (2011). A framework for variation discovery and genotyping using next-generation DNA sequencing data. Nat. Genet..

[30] Reumers J., De Rijk P., Zhao H., Liekens A., Smeets D., Cleary J., Van Loo P., Van Den Bossche M., Catthoor K., Sabbe B. (2012). Optimized filtering reduces the error rate in detecting genomic variants by short-read sequencing. Nat. Biotechnol..

[31] Liu D., Ma C., Hong W., Huang L., Liu M., Liu H., Zeng H., Deng D., Xin H., Song J. (2014). Construction and analysis of high-density linkage map using high-throughput sequencing data. PLoS ONE.

[32] Huang X., Feng Q., Qian Q., Zhao Q., Wang L., Wang A., Guan J., Fan D., Weng Q., Huang T. (2009). High-throughput genotyping by whole-genome resequencing. Genome Res..

[33] Van Os H., Stam P., Visser R.G., van Eck H.J. (2005). SMOOTH: A statistical method for successful removal of genotyping errors from high-density genetic linkage data. Theor. Appl. Genet..

[34] Tang H., Zhang X., Miao C., Zhang J., Ming R., Schnable J.C., Schnable P.S., Lyons E., Lu J. (2015). ALLMAPS: Robust scaffold ordering based on multiple maps. Genome Biol..

[35] Li B., Lu X., Dou J., Aslam A., Gao L., Zhao S., He N., Liu W. (2018). Construction of a high-density genetic map and mapping of fruit traits in watermelon (*Citrullus lanatus* L.) based on whole-genome resequencing. Int. J. Mol. Sci..

[36] Demo B.K. (2007). Development of Intervarietal Substitution Lines in *Brassica napus* L. Using Marker Assisted Selection and Mapping of QTL for Agronomically Important Traits. Ph.D. Thesis.

[37] Yadava S.K., Ramchiary N. (2022). Molecular Linkage Mapping in *Brassica juncea*: Founding the Basis for Marker-Assisted Selection. The Brassica juncea Genome.

[38] Boopathi N.M., Boopathi N.M. (2020). Linkage map construction. Genetic Mapping and Marker Assisted Selection: Basics, Practice and Benefits.

[39] Yu H., Wang J., Sheng X., Zhao Z., Shen Y., Branca F., Gu H. (2019). Construction of a high-density genetic map and identification of loci controlling purple sepal trait of flower head in *Brassica oleracea* L. *italica*. BMC Plant Biol..

[40] MPerez-de-Castro A., Vilanova S., Cañizares J., Pascual L., MBlanca J., JDiez M., Prohens J., Picó B. (2012). Application of genomic tools in plant breeding. Curr. Genom..

[41] Lim J.-H., Yang H.-J., Jung K.-H., Yoo S.-C., Paek N.-C. (2014). Quantitative trait locus mapping and candidate gene analysis for plant architecture traits using whole genome re-sequencing in rice. Mol. Cells.

[42] Xu X., Zeng L., Tao Y., Vuong T., Wan J., Boerma R., Noe J., Li Z., Finnerty S., Pathan S.M. (2013). Pinpointing genes underlying the quantitative trait loci for root-knot nematode resistance in palaeopolyploid soybean by whole genome resequencing. Proc. Natl. Acad. Sci. USA.

[43] Scheben A., Batley J., Edwards D. (2017). Genotyping-by-sequencing approaches to characterize crop genomes: Choosing the right tool for the right application. Plant Biotechnol. J..

[44] Torkamaneh D., Boyle B., Belzile F. (2018). Efficient genome-wide genotyping strategies and data integration in crop plants. Theor. Appl. Genet..

[45] Le Nguyen K., Grondin A., Courtois B., Gantet P. (2019). Next-generation sequencing accelerates crop gene discovery. Trends Plant Sci..

