# Supplementary material for: Construction and Application of an F1-Derived Doubled-Haploid Population and High-Density Genetic Map for Ornamental Kale Breeding
Source: Genes (Basel). 2023 Nov 20;14(11):2104. doi: 10.3390/genes14112104 (PMC10670981; doi:10.3390/genes14112104)
Supplement: Supplementary file 1 [file genes-14-02104-s001.zip › genes-2678810-supplementary.pdf]

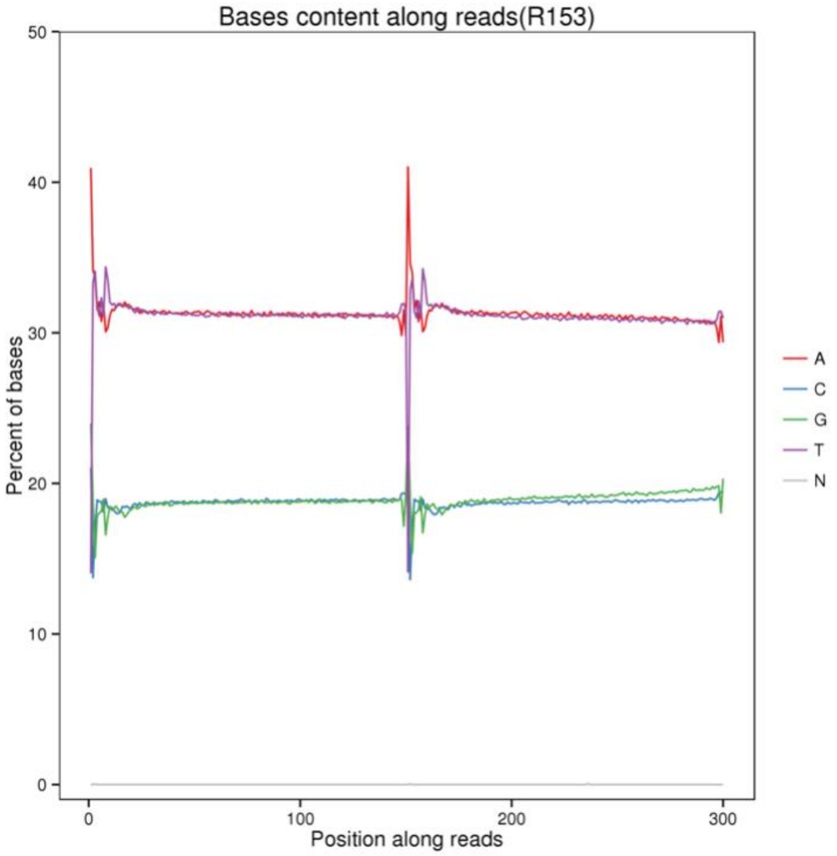

Supplementary Figure S1 Base ratio distribution for the female parental line 05-DH-65.

Supplementary Table S1 Coverage depth and coverage ratio of the sequencing data

Sample	Average depth	Average coverage ratio 1X (%)	Average coverage ratio 5X (%)	Average coverage ratio 10X(%)
05-DH-65	64.00	93.73	91.50	89.97
06-DH-71	34.00	93.37	90.32	87.97
F1DH offspring	2.60	72.55	16.24	3.01

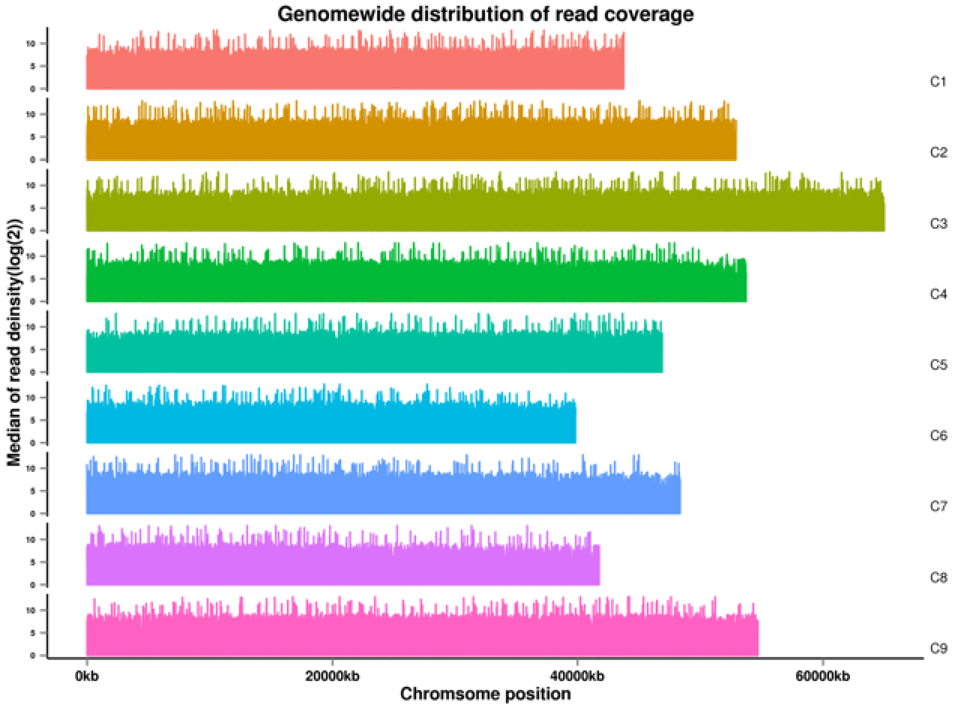

Supplementary Figure S2 Coverage depth distribution on chromosomes of the female parental line. The *x*-axis represents the position on each chromosome. The *y*-axis presents the $\log_2$ value of the coverage depth.

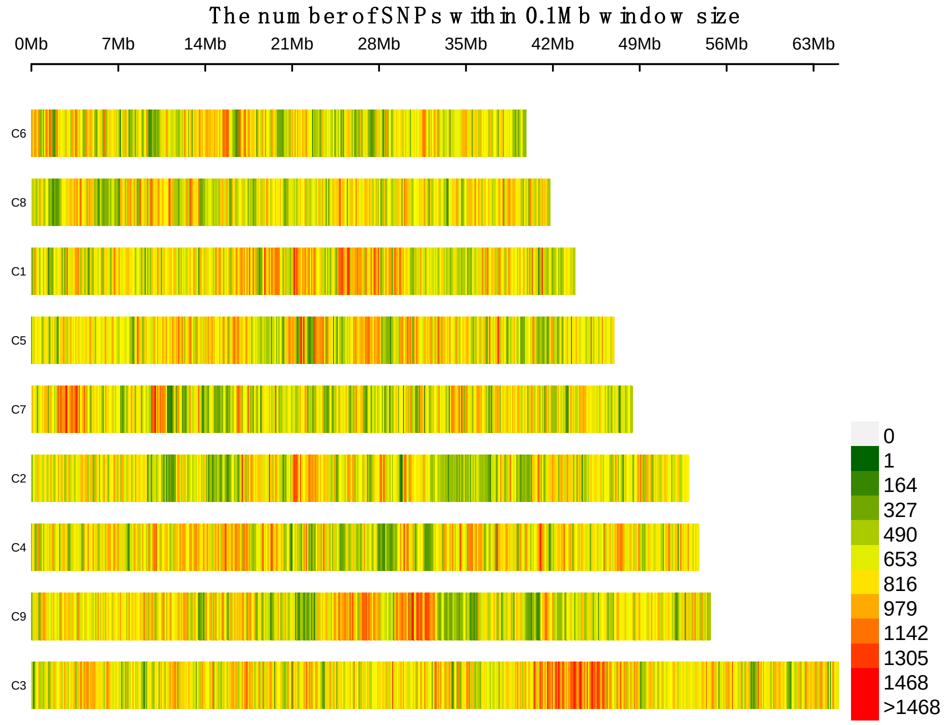

Supplementary Figure S3 Heatmap of the distribution of SNPs across the nine chromosomes of the ornamental kale genome. A window size of 0.1 Mb was selected on the genome to count the number of SNPs. The color ranges from green to red indicating the density of SNPs from low to high.

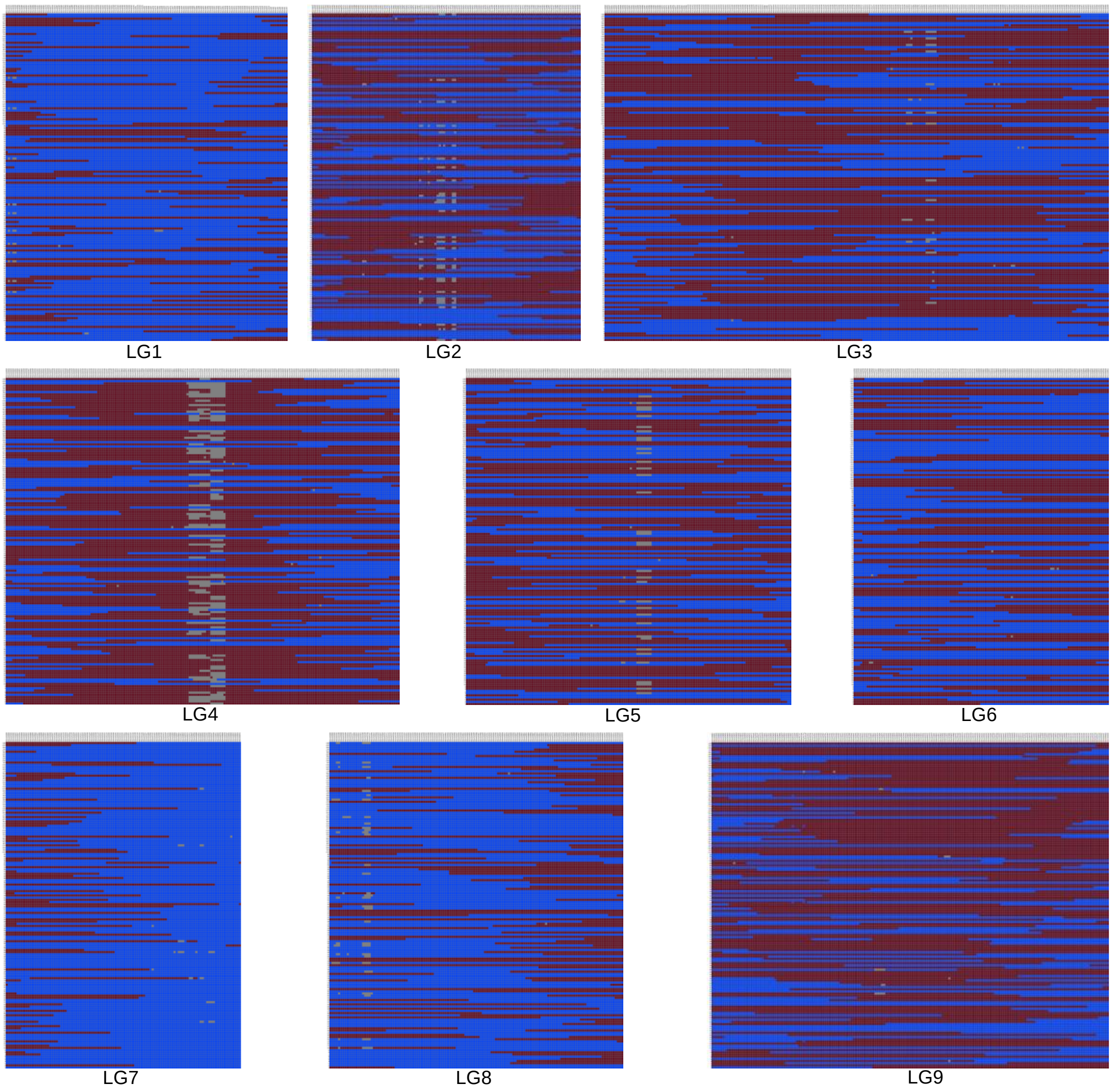

Supplementary Figure S4 Haplotype map of the genetic map. Each row represents a marker, arranged in the order in the linkage map. Each column represents one sample. Red represents the female parent, blue represents the male parent, and gray indicates missing data. Where the color changes in a column is the location of a recombination event.

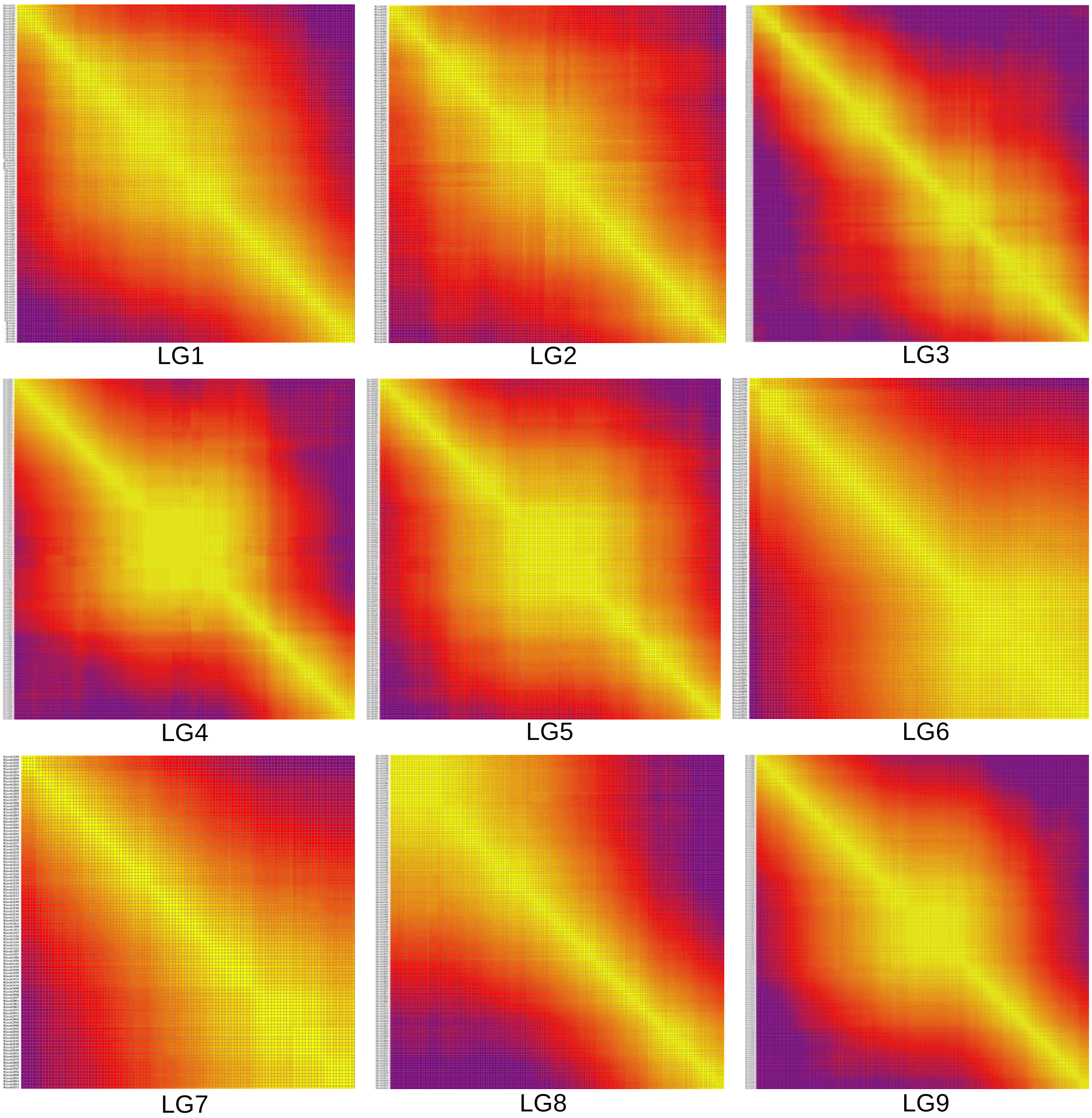

Supplementary Figure S5 Heatmap of the genetic map. The x and y axes represent markers. The order of markers in a row and column are arranged according to their genetic distance. Each row and column is arranged in the order of markers on the map. Each cell represents the recombination rate between two markers, with the color gradient from yellow to red to purple indicating a progression from lower to higher recombination rates. Markers that are closer to each other have a lower recombination rate and are closer to yellow in color, while markers that are further apart have a higher recombination rate, indicated by a color closer to purple.

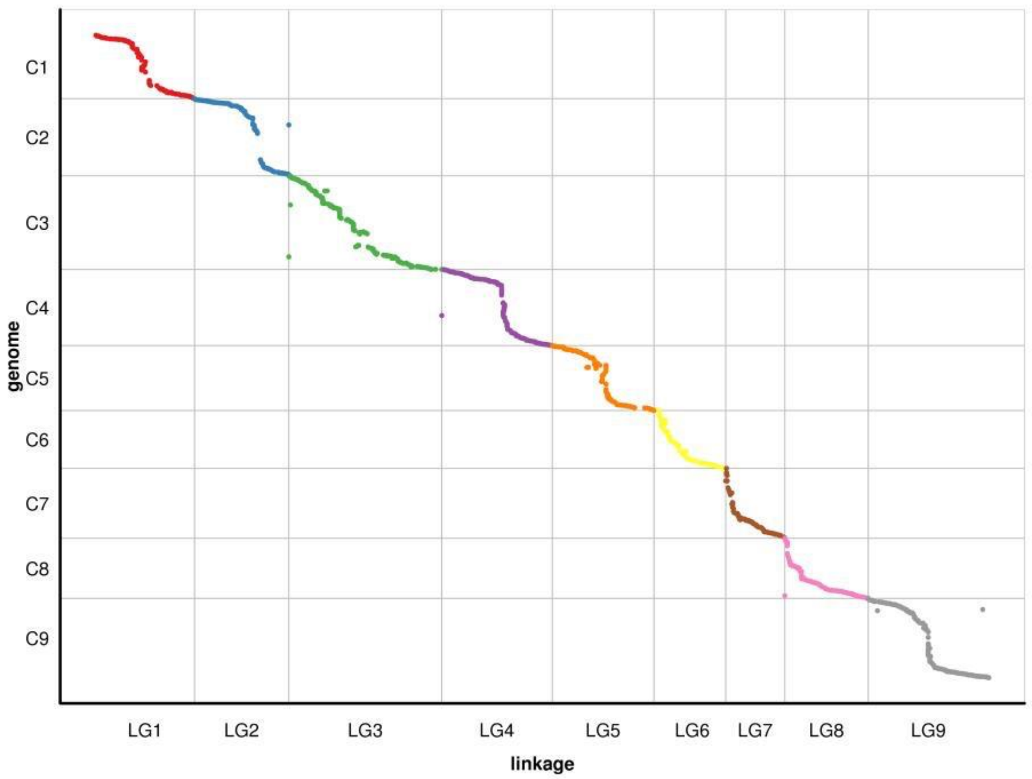

Supplementary Figure S6 Relationship between genetic and physical positions for each chromosome. The x-axis represents the genetic distance on the genetic map, and the y-axis represents the physical position on the genome. The different colors only indicated the different LGs.

Table S2. Spearman correlation coefficients between the genetic and physical positions of each linkage group (LG).

LG ID	Spearman
LG1	0.9987
LG2	0.9926
LG3	0.9778
LG4	0.9816
LG5	0.9842
LG6	0.9976
LG7	0.9981
LG8	0.9641
LG9	0.9803

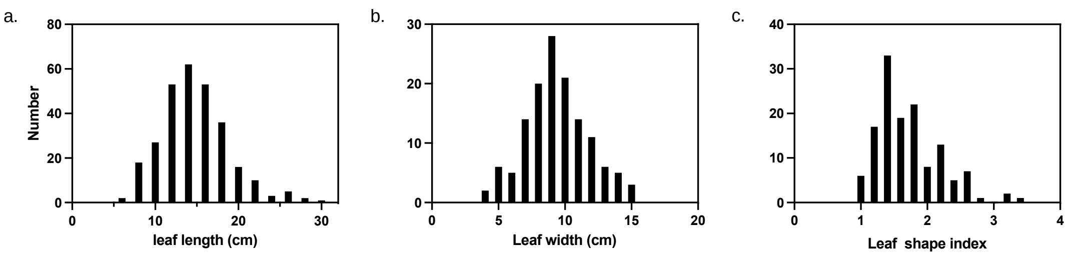

Supplementary Figure S7 Histograms depicting the distribution of leaf morphological traits in a plant population. a, b, c, display the leaf length, leaf width, leaf shape index distribution, respectively.
